# Vascular Rings as a Cause of Respiratory Difficulties

**DOI:** 10.3390/life16101736

**Published:** 2026-10-16

**Authors:** Jakub Šulov, Filip Olekšák, Jarmila Vojtková, Pavlína Balázsová, Alžbeta Soršáková, Otília Petrovičová, Anna Durdikova, Peter Durdik, Miloš Jeseňák

**Affiliations:** 1 Department of Pediatrics and Adolescent Medicine, University Hospital Martin, Kollárová 2, 036 59 Martin, Slovakia; deti.kdad@unm.sk (J.Š.); paja.sucha@gmail.com (P.B.); majtanova23@uniba.sk (A.S.); petrovicov22@uniba.sk (O.P.); anna.durdikova@uniba.sk (A.D.); peter.durdik@uniba.sk (P.D.); milos.jesenak@uniba.sk (M.J.); 2 Jessenius Faculty of Medicine in Martin, Comenius University in Bratislava, Malá Hora 4A, 036 01 Martin, Slovakia

**Keywords:** vascular rings, echocardiography, respiratory difficulties, surgical repair, pediatric patients

## Abstract

**Background**: Vascular rings are rare congenital developmental anomalies of the cardiovascular system that present with variable respiratory and gastrointestinal symptoms often refractory to what is considered a standard treatment. The aim of our study was to describe the clinical presentation, diagnostic pathway, and management of children referred to a tertiary pediatric department for unexplained respiratory symptoms in whom a vascular ring was confirmed, with particular attention to the role of echocardiography in raising the initial suspicion, and to compare our findings with published cohorts. **Methods**: We performed a retrospective descriptive case series of patients (*n* = 7) diagnosed at the Department of Pediatrics and Adolescent Medicine, Jessenius Faculty of Medicine in Martin, Comenius University in Bratislava, University Hospital Martin, between 2021 and 2025. **Results**: The median age at onset of clinical symptoms was 30 days (interquartile range [IQR] 1–120 days). The median age at definitive diagnosis was 14.5 months (IQR 4.3–94.0 months). A genetic abnormality was observed in 3 patients (42.9%), and a positive family history of congenital heart defects was present in 4 (57.1%). All patients (*n* = 7) were symptomatic at the time of definitive diagnosis. We observed double aortic arch (DAA) in 2 patients (28.6%), right aortic arch with aberrant left subclavian artery (RAA + ALSA) in 4 (57.1%), and aberrant right subclavian artery (ARSA) in 1 (14.3%). One patient with RAA + ALSA had a concurrent pulmonary artery sling (PAS). In 6 patients (85.7%), the initial suspicion was raised by transthoracic or prenatal echocardiography. Six patients (85.7%) underwent surgical repair at a median age of 10.5 months (IQR 4.1–20.1 months), while in one case the risk–benefit ratio of surgery is being evaluated. **Conclusions**: In this series, echocardiography was a useful non-invasive modality for raising the initial suspicion, whereas CT angiography was required to define the vascular anatomy; the majority of patients were definitively diagnosed and treated within the first two years of life. In our cohort, we also identified a rare combination of a vascular ring (RAA + ALSA) and a pulmonary artery sling (PAS) in a single patient.

## 1. Introduction

Respiratory difficulties are among the most common reasons for seeking pediatric care, and congenital cardiovascular anomalies represent one of their less common but important causes. Vascular rings and slings are rare congenital anomalies of the aortic arch and pulmonary arteries, affecting approximately 1 in 10,000 live-born children [1]. Persistence of respiratory and gastrointestinal symptoms in infants and young children that are refractory to conventional therapy should raise suspicion of a vascular ring [2].

The term vascular ring refers to congenital anomalies of the aortic arch that compress the trachea, esophagus, or both, resulting in typical respiratory and gastrointestinal symptoms [3]. The term was introduced by Gross, who in 1945 described the first surgical treatment of a double aortic arch [4].

The basis of vascular ring formation lies in the abnormal fusion and regression of the six pairs of primitive aortic arches, from which, under normal circumstances, the definitive aortic arch, the major vessels arising from the arch, the ductus arteriosus, and the proximal segments of the pulmonary arteries develop [5].

Tracheal compression leads to the typical presentation of labored breathing, stridor, and a barking cough in children but may also result in frequent recurrences of respiratory infections, episodes of cyanosis, or apneic pauses. Esophageal compression manifests as feeding difficulties, dysphagia, or recurrent vomiting [6]. Respiratory symptoms predominate; for example, in double aortic arch (DAA), respiratory symptoms occur in 90% of cases and gastrointestinal symptoms in 40% of cases [7]. In most cases, symptoms manifest during the first year of life; however, in many cases, the anatomical abnormality remains asymptomatic [3].

According to the International Congenital Heart Surgery Nomenclature and Database Committee, vascular rings are classified as complete and incomplete rings [8]. Complete vascular rings completely encircle the trachea and esophagus. The most common examples are DAA and right aortic arch with aberrant left subclavian artery (RAA + ALSA), in which the ring is completed by a left-sided ligamentum arteriosum. Incomplete vascular rings cause only partial compression and include pulmonary artery sling (PAS), also referred to as a vascular sling, aberrant right subclavian artery (ARSA), and innominate artery compression syndrome [8].

Initial suspicion of a vascular ring depends on the pediatrician’s awareness and a thorough history of respiratory and gastrointestinal symptoms that respond inadequately to conventional treatment. There is no universally accepted diagnostic algorithm; however, several studies have proposed different diagnostic approaches [3,9]. Commonly used imaging modalities include chest X-ray, barium esophagography, CT and MRI angiography, echocardiography, and bronchoscopy. A newer diagnostic modality that has significantly influenced patient management is prenatal echocardiography [10].

Treatment of vascular rings has traditionally been reserved for symptomatic patients. It consists of surgical division of the structure causing tracheal and esophageal compression. Early intervention is preferred to prevent organ damage resulting from recurrent infections and airway obstruction. It is generally well established that these procedures have excellent therapeutic outcomes with a low incidence of complications [11]. With the increasing use of prenatal diagnosis, the number of asymptomatic patients is rising, and early intervention is increasingly discussed to prevent complications of prolonged compression, as surgery at an older age may be associated with a higher risk [2].

Despite their clinical relevance, vascular rings are frequently overlooked. Their symptoms are nonspecific and often mimic far more common conditions, such as recurrent respiratory infections, bronchial asthma, or gastroesophageal reflux, which may lead to repeated ineffective treatment and a substantial diagnostic delay [2,9]. Many published cohorts are surgical series focusing primarily on operative techniques and outcomes [11], whereas data describing these patients from the perspective of a general pediatric department, where children usually present first with unexplained respiratory symptoms, are limited. In Slovakia, aortic arch anomalies have previously been characterized mainly from a morphological perspective in a 20-year cohort of the national pediatric cardiac center [12]. Recent data focusing on the clinical presentation and diagnostic pathway in children referred for unexplained respiratory symptoms are scarce. Moreover, the optimal diagnostic pathway remains a matter of debate. Traditional algorithms rely on chest X-ray and barium esophagography as first-line investigations [9], while the role of echocardiography as an initial screening tool in symptomatic children has not been clearly established. Better recognition of early clinical clues and an effective diagnostic sequence could shorten the time to diagnosis and allow timely surgical treatment.

Therefore, the aim of this study was to analyze children referred to our tertiary pediatric department for the differential diagnosis of respiratory symptoms of unclear etiology, in whom a vascular ring was subsequently confirmed. Specifically, we aimed to characterize the timing and spectrum of respiratory and gastrointestinal symptoms, associated cardiac and genetic abnormalities, and the age at definitive diagnosis; to evaluate the contribution of individual imaging modalities, particularly echocardiography, to raising the initial suspicion of a vascular ring; and to describe surgical management, its timing, and outcomes. Our findings are compared with previously published cohorts in order to identify practical clinical clues that may facilitate earlier recognition of vascular rings in everyday pediatric practice.

## 2. Materials and Methods

### 2.1. Study Design and Patients

In this descriptive case series, we retrospectively analyzed all patients (*n* = 7) who were referred to the Department of Pediatrics and Adolescent Medicine, Jessenius Faculty of Medicine in Martin, Comenius University in Bratislava, University Hospital Martin, Slovakia, from regional hospitals for the differential diagnosis of respiratory symptoms of unclear etiology (and in one case with prenatal suspicion for a vascular ring) and in whom the diagnosis of a vascular ring was definitively confirmed. No potentially eligible patient was excluded. Data were collected for the period from 2021 to 2025. Patients under follow-up during this period but who had been diagnosed before 2021 were not included in the study. Patients in whom the diagnosis of a vascular ring was not confirmed were excluded. The diagnosis was confirmed based on the analysis of imaging findings by a board-certified radiologist and pediatric cardiologist. Chest X-ray, CT angiography of the chest and great vessels, transthoracic echocardiography, and bronchofibroscopy were analyzed. Individual types of vascular rings were classified according to the International Congenital Heart Surgery Nomenclature and Database Committee [8]. All patients remain under follow-up at the cardiology outpatient clinic to date, with the frequency of visits depending on the time elapsed since surgery and their clinical status.

The parameters assessed in the patients included sex, gestational age at birth, age at onset of clinical symptoms, age at definitive diagnosis of the vascular ring, associated cardiovascular abnormalities and genetic syndromes, family history of cardiovascular disease, clinical symptoms, imaging modalities used to raise suspicion of the diagnosis and the type of confirmatory imaging modality, type of vascular ring, type of treatment, age at treatment, diagnosis-to-surgery interval, treatment outcome, and possible surgery complications.

### 2.2. Data Analysis

Patient information and data were obtained from the electronic hospital information system NIS MEDEA (STAPRO Slovensko s.r.o., Košice, Slovakia, version 3.60.A.01). Patients were identified based on a search of ICD-10 codes for the individual types of vascular rings and slings (Q25.4, Q25.7, Q25.8, Q27.8). Through a retrospective review of patients’ medical records, we analyzed the demographic parameters, symptoms, and examinations described above. Imaging data were analyzed using TOMOcon PACS software (TatraMed software s.r.o., Bratislava, Slovakia, version 15.0.0.7381). Categorical data were presented as absolute numbers and percentages of the patient cohort. Given the small sample size and the skewed distribution of most continuous variables, continuous data were reported as medians with interquartile ranges (IQR; first to third quartile, calculated using the inclusive method) and, where informative, ranges. Owing to the small sample size, no inferential statistical testing was performed. All collected data were processed using Microsoft Excel (Microsoft Corp., Redmond, WA, USA, version 2608, build 20326.20232).

### 2.3. Ethics

All procedures within the study complied with the ethical standards of the institutional and national research committees and with the principles of the Declaration of Helsinki (1964 and its later amendments). Patient consent was waived due to the study’s retrospective nature and covered retrospective use of pediatric clinical and imaging data. All scans were anonymized. The requirement for separate consent for publication was waived by the Ethics Committee in accordance with institutional policy. The study protocol was approved by the Ethics Committee of the Jessenius Faculty of Medicine in Martin, Comenius University in Bratislava (Slovak Republic; approval no. EK 10/2026; date of approval: 29 January 2026).

## 3. Results

### 3.1. Demographics

A total of 7 patients with a confirmed diagnosis of a vascular ring (male = 3, 42.9%) were analyzed over the period from 2021 to 2025. Detailed patient-level data, including clinical characteristics, imaging modalities, surgical procedures, complications, outcomes, and duration of follow-up, are presented in Supplementary Table S1. The median age at onset of clinical symptoms was 30 days (IQR 1–120 days; range 1–5170 days). The wide range reflects one patient in whom the symptoms started in adolescence (at 14 years of age). Three children (42.9%) presented with symptoms from the first day of life. The median age at definitive diagnosis, confirmed by CT angiography, was 14.5 months (IQR 4.3–94.0 months; range 1.25–185 months). The wide range reflects two patients diagnosed in adolescence (at 15 and 14 years of age). The median gestational age at birth was 38 weeks (IQR 36.5–39 weeks); two patients (28.6%) were born preterm, at 32 and 36 weeks of gestation. Most patients were eutrophic at birth (median birth weight 2600 g, IQR 2048–3205 g); in two cases, the child was born small for gestational age (see Table 1). Genetic testing was performed in four patients; Down syndrome was confirmed in one (14.3%) and DiGeorge syndrome in two (28.6%). In one patient, suspected DiGeorge syndrome was not confirmed. Family history revealed a cardiovascular burden in 4 cases (57.1%), including three patients with a positive family history of early death of a close family member due to congenital heart disease (CHD).

### 3.2. Symptomatology

The majority of patients presented with a typical combination of predominantly respiratory symptoms and less frequent gastrointestinal symptoms. All patients were symptomatic. One patient remained asymptomatic for a prolonged period, with the first symptoms appearing at the age of 14 years. Until then, the patient had only been under follow-up for a suspected and unspecified abnormality of the aortic arch. The most common symptoms were cough and dyspnea (*n* = 6, 85.7%). Other frequent respiratory symptoms included stridor and recurrent lower respiratory tract infections (*n* = 5, 71.4%), as well as cyanosis (*n* = 4, 57.1%). Apneic episodes were less frequently observed (*n* = 2, 28.6%), and, notably, wheezing was not reported in any patient. Regarding gastrointestinal symptoms, dysphagia (*n* = 5, 71.4%) and recurrent vomiting (*n* = 4, 57.1%) predominated, while failure to thrive (*n* = 2, 28.6%) and prolonged feeding (*n* = 1, 14.3%) were less common (see Table 2).

### 3.3. Diagnostic Work-Up

During the diagnostic process, several imaging examinations were performed based on a suspected clinical presentation suggestive of an atypical etiology of respiratory and gastrointestinal symptoms. In the majority of cases, standard transthoracic echocardiography was selected as the first-line imaging modality (*n* = 5, 71.4%). In only one case, bronchofibroscopy was performed as the initial examination due to the need for detailed assessment of the airway anatomy in a patient with a previously known congenital laryngomalacia. Definitive confirmation of the diagnosis was obtained by CT angiography of the chest in all cases (*n* = 7, 100%). The sequence of imaging examinations in individual patients is shown in Supplementary Table S1. Echocardiography was performed in all patients, chest X-ray in five (71.4%), and bronchofibroscopy in six patients (85.7%), in whom it most often followed CT angiography (*n* = 5). Only one patient in our cohort was diagnosed prenatally. Although a chest X-ray was performed in several patients, it was insufficient to raise suspicion of a vascular ring.

### 3.4. Vascular Rings, Slings, and Other Cardiac Findings

**Figure 1:**
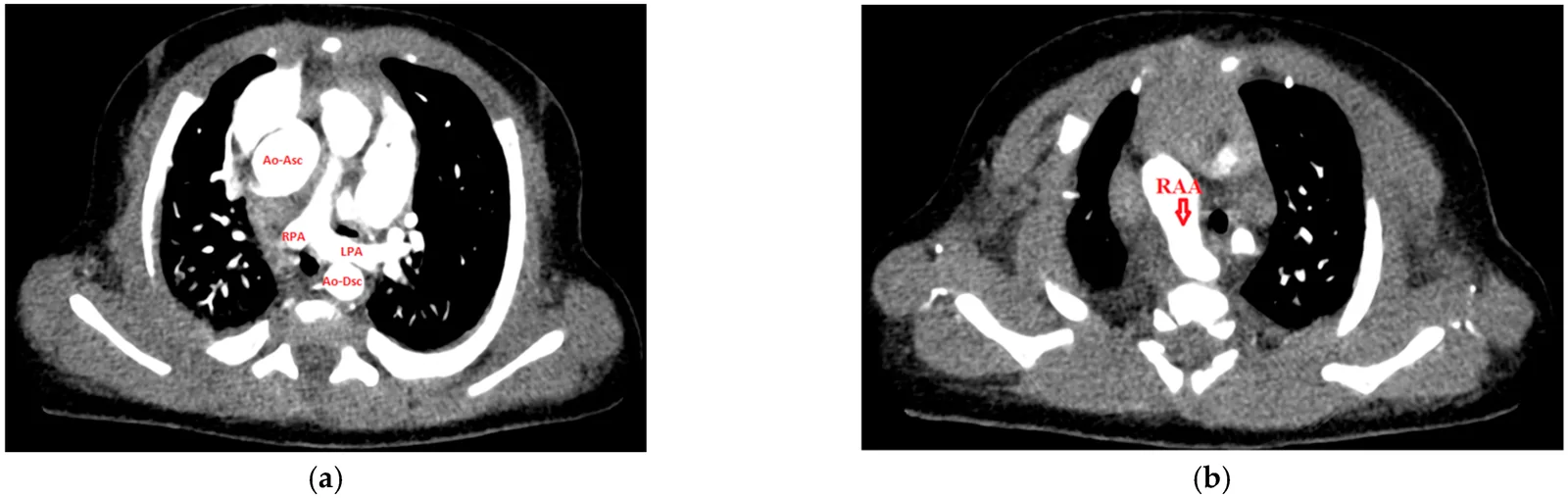
CT angiography findings in a patient with a combination of a vascular ring and a pulmonary artery sling. In image (**a**), a scan at the level of the pulmonary artery sling (PAS) demonstrates the left pulmonary artery (LPA) arising more distally, directly from the right pulmonary artery (RPA). Partial narrowing of the left bronchus due to LPA compression can be observed. Image (**b**) demonstrates a portion of the vascular ring, namely the right aortic arch (RAA), with its position to the right of the trachea, which represents one of the criteria for determining the laterality of the aortic arch. Ao-Asc—ascending aorta; Ao-Dsc—descending aorta.

In our cohort, the following types of vascular rings were observed. A double aortic arch (DAA) was identified in 2 cases (28.6%), with right-sided branch dominance in one case and no side dominance in the other case. A right aortic arch with an aberrant left subclavian artery (RAA + ALSA) was observed in 4 cases (57.1%); in all of them, the ring was completed by a left-sided ligamentum arteriosum. This anomaly is frequently associated with a Kommerell diverticulum, which was present in all cases of RAA + ALSA (*n* = 4, 100%). Aberrant right subclavian artery (ARSA) was observed in one case (14.3%). We also recorded one rare case of a complete vascular ring (RAA + ALSA) coexisting with a pulmonary artery sling (see Figure 1). Associated cardiac anomalies were found in four patients (57.1%). The most common was a bicuspid aortic valve (*n* = 2, 28.6%); other congenital heart defects occurred individually. One case of patent ductus arteriosus (PDA) was identified. In the patient with the combination of PAS and RAA + ALSA, ventricular and atrial septal defects, as well as a left-sided superior vena cava, were also described.

### 3.5. Management and Timing of Surgery

Six of the seven patients (85.7%) underwent surgical treatment. In one patient with late onset of symptoms (the only case of ARSA), the risk–benefit ratio of surgery is being carefully evaluated. Surgical treatment was aimed at eliminating the cause of tracheal and esophageal compression. In cases of DAA with known side dominance, the non-dominant aortic arch was resected. In the case without side dominance, the right aortic arch was resected. The ligamentum arteriosum was also divided. Correction of RAA + ALSA consisted of division of the ligamentum arteriosum together with reimplantation or mobilization of the ALSA. In the aforementioned rare combination of RAA + ALSA and PAS, division of the ligamentum arteriosum and reimplantation of the left pulmonary artery into the main pulmonary artery were performed, together with tracheoplasty for the associated ring–sling complex (complete cartilaginous tracheal rings).

Owing to the small number of patients, the data were influenced by one patient who was diagnosed and treated at an advanced age (diagnosed at 15 years and operated on at almost 17 years of age), which is reflected in the wide ranges of the following parameters. The median age at surgery was 10.5 months (IQR 4.1–20.1 months; range 1.5–202 months). Another parameter assessed was the diagnosis-to-surgery interval, defined as the time from definitive diagnosis of the vascular ring to surgical intervention. The median diagnosis-to-surgery interval was 10 days (IQR 7–160.8 days; range 7–540 days).

### 3.6. Outcome and Follow-Up

Postoperative complications were recorded in 2 of 6 operated patients (33.3%), specifically chylothorax following thoracic duct ligation and thrombosis of the left subclavian artery following its mobilization during the procedure. The treatment outcome was also favorable in the majority of cases. In one case, respiratory symptoms improved only partially due to pre-existing tracheolaryngomalacia requiring a tracheostomy cannula. In the remaining patients, symptoms almost completely resolved, with only minimal residual symptoms in the form of occasional stridor. All operated patients remain under regular follow-up, with a median postoperative follow-up of 16 months (IQR 15.3–17.5 months; range 7–65 months).

## 4. Discussion

In our study, we retrospectively analyzed individual cases of patients with vascular rings, from the clinical onset of their symptoms through to eventual treatment.

The literature generally reports an early onset of clinical symptoms in children, usually during the first 6 months of life; comparable studies reported a median age at symptom onset ranging from 4 to 7.65 months [10,13,14]. In our cohort, the median age at symptom onset was considerably earlier, at 30 days (IQR 1–120 days), with three patients (42.9%) manifesting clinically from the day of birth. Regarding the timing of symptom onset according to the individual types of vascular rings, DAA is considered to manifest earliest (at 3–6 months of life), while RAA + ALSA may present somewhat later [15,16]. This pattern was not observed in our cohort: the two patients with DAA became symptomatic on the first day of life and at 2 months of age, respectively, whereas three of the four patients with RAA + ALSA were symptomatic from birth or within the first month of life. Similarly, Bertone et al. reported earlier symptom onset in symptomatic patients with RAA + ALSA than in those with DAA (median 1 day vs. 9 months) [10]. However, the small number of patients in our cohort does not allow a meaningful comparison between individual ring types. We also observed a late manifestation of an incomplete vascular ring (ARSA) in one patient, who became symptomatic in adolescence. PAS frequently presents already in neonates and young infants, which was consistent with our only case of PAS [17].

The median age at definitive diagnosis in our cohort was 14.5 months (IQR 4.3–94.0 months), which is slightly later than in most comparable studies, mainly owing to two patients diagnosed in adolescence; on the other hand, three patients (42.9%) were diagnosed within the first 5 months of life. Other studies report varying ages and often broad ranges; the median age at diagnosis is generally reported to range from 5.5 to 13 months of life [14,16]. The emerging trend toward improved prenatal diagnosis may significantly influence the management of these patients in the future.

Vascular rings are frequently associated with various genetic abnormalities. The most commonly documented include 22q11.2 deletion syndrome (DiGeorge syndrome), trisomy 21 (Down syndrome), CHARGE syndrome, trisomy 18 (Edwards syndrome), and others. In a retrospective population-based multicenter study from the United Kingdom, a genetic abnormality was identified in at least 7.6% of fetuses prenatally diagnosed with RAA or DAA, most frequently 22q11.2 microdeletion [18]. In our cohort, a genetic abnormality was identified in 3 cases (42.9%), including 2 cases of DiGeorge syndrome (28.6%) and 1 case of Down syndrome (14.3%), representing a considerably higher proportion, likely reflecting the selection of symptomatic patients referred to a tertiary center and the small sample size. The predominance of DiGeorge syndrome was consistent with the current literature.

In our cohort, all patients were symptomatic at the time of definitive diagnosis, although one patient (the only case of ARSA) had been asymptomatic for most of their life and presented with only slight dyspnea during physical exercise. The absence of asymptomatic patients reflects the selection of our cohort, which consisted of patients referred for respiratory symptoms, and contrasts with older studies reporting asymptomatic cases in 20–35% of patients [19,20,21]. With improvements in prenatal diagnosis, the proportion of asymptomatic patients has further increased in more recent studies; for example, in a cohort of 115 patients, only 17.5% were symptomatic [10]. The clinical symptoms in our cohort were consistent with previous observations, with respiratory symptoms predominating. Among respiratory symptoms, cough and dyspnea were most common (*n* = 6, 85.7%), followed by stridor and recurrent lower respiratory tract infections (*n* = 5, 71.4%). The most common gastrointestinal symptoms were varying degrees of dysphagia (*n* = 5, 71.4%) and recurrent vomiting (*n* = 4, 57.1%). We did not observe differences in the clinical presentation between the individual types of vascular rings. This is probably due to the small size of our cohort.

Regarding the type of vascular ring, available studies indicate a predominance of complete vascular rings (DAA, RAA + ALSA, etc.), accounting for approximately 73% of cases, compared with incomplete rings (PAS, ARSA, etc.) in 27% of cases. The most common type is RAA + ALSA, accounting for 39–77% of cases, followed by DAA in 20–33% and PAS in 3–26% of cases. Other types of vascular rings are considerably less common [11,21,22]. Our cohort reflected the predominance of RAA + ALSA in 57.1% of cases (*n* = 4) compared with DAA in 28.6% of cases (*n* = 2) and ARSA in 14.3% of cases (*n* = 1). We observed one case of PAS; however, it was not included separately in the aforementioned statistics because it occurred in combination with a vascular ring (RAA + ALSA) in a single patient (see Figure 1). This patient had Down syndrome and was one month old at the time of diagnosis. The coexistence of a vascular ring and a pulmonary artery sling is extremely rare in the literature and has been described only in individual case reports [17,23,24,25,26]. Another important finding in this patient was the ring–sling complex, i.e., the combination of PAS with complete (circumferential) cartilaginous tracheal rings, which further accentuates airway obstruction and is generally associated with a higher risk of mortality [9]. The patient also had the greatest number of additional cardiac findings in our cohort, namely a ventricular septal defect, atrial septal defect, and left-sided superior vena cava. Other cardiac findings observed in our cohort included bicuspid aortic valve (BAV) and PDA. Overall, associated cardiac abnormalities were observed in 57.1% (*n* = 4) of cases, which is broadly consistent with a study of 106 patients that identified an additional cardiac defect in 48% of cases. The most common findings included ventricular septal defect, tetralogy of Fallot, atrioventricular septal defect, atrial septal defect, PDA, and others [27].

There is no universally established diagnostic algorithm for confirming the diagnosis of a vascular ring. In 2015, Licari et al. proposed a diagnostic approach based on frontal and lateral chest X-rays together with barium esophagography as the first step in raising suspicion of a vascular ring. In cases with positive findings, they recommended CT or MR angiography or bronchofibroscopy for confirmation. Echocardiography was considered complementary for the assessment of associated cardiac abnormalities [9]. In contrast to this proposed approach, echocardiography was used as the primary imaging modality for raising suspicion of a vascular ring in our cohort (transthoracic echocardiography, *n* = 5, 71.4% of cases, and prenatal echocardiography, *n* = 1, 14.3% of cases), emphasizing the importance of thorough cardiological evaluation when a non-pulmonary etiology of prolonged respiratory symptoms is suspected [28]. Thorough echocardiography in cooperative children (sedated if necessary) may serve as a red-flag tool in symptomatic patients. Given its noninvasive nature, its diagnostic value should be formally validated before it can be incorporated into clinical guidelines. In one case, suspicion of a vascular ring was raised by bronchofibroscopy performed to assess the extent of congenital laryngomalacia. Although many patients underwent chest X-rays, none of these examinations raised suspicion of a vascular ring; however, it should be noted that only frontal views were obtained. Barium esophagography, which was more commonly used historically, was not performed in our cohort. The principal modality for definitive confirmation of the diagnosis was CT angiography (CTAG) (*n* = 7, 100%).

Vascular rings are increasingly being diagnosed prenatally. Worhunsky et al. investigated the impact of prenatal diagnosis on the management of this condition and proposed a new diagnostic approach for these patients. In general, they recommend reassessment of the prenatal finding after birth. Depending on the presence of symptoms, CTAG should be performed, followed by surgical intervention. In asymptomatic patients, they recommend scheduled CTAG at 2–4 months of age. If significant tracheal obstruction is identified, surgical intervention is recommended. Even in the presence of an asymptomatic state and non-significant tracheal obstruction, elective surgery may be considered, with the anticipated timing of surgery determined according to the type of vascular ring [3]. In our study, only one patient was diagnosed prenatally. Despite confirmation of airway compression by both CTAG and bronchofibroscopy, the patient underwent surgery relatively late, at 21.5 months of age (definitive diagnosis by CTAG at 14.5 months, followed by surgery 210 days later).

Most symptomatic patients in our cohort (*n* = 6, 85.7%) underwent surgical treatment. The age at definitive diagnosis in our study was skewed by two patients diagnosed during adolescence. In these patients, we encountered the relatively frequently documented phenomenon of incorrectly diagnosed and treated bronchial asthma in patients with vascular rings. Cases of asthma treatment lasting several decades followed by late diagnosis of a vascular ring have also been reported [29]. One of these late-diagnosed patients underwent surgery, while in the other, the risk–benefit ratio of surgery is still being evaluated because of mild symptoms. The median age at surgical treatment in our cohort was 10.5 months (IQR 4.1–20.1 months), which is comparable with larger patient cohorts reporting median ages at surgery of approximately 9–11 months [22,30]. In our study, we also assessed the diagnosis-to-surgery interval. The median diagnosis-to-surgery interval was 10 days (IQR 7–160.8 days). This interval is not frequently reported in other studies; Suh et al. reported a median interval of 6 days between diagnosis and operation [19], which is comparable to our findings. However, a trend toward early surgical intervention in younger children and a longer interval in cases of late diagnosis has been observed [31]. Surgical treatment of vascular rings is considered safe, with favorable treatment outcomes and a low incidence of perioperative or postoperative complications. The overall complication rate is generally reported to range from 14 to 18% of cases [22,32]. In our cohort, all operated patients survived; complications occurred in two of six operated patients (33.3%), representing a higher rate, most likely due to the small sample size. The majority of our patients demonstrated favorable treatment outcomes, with minimal residual symptoms.

In recent years, increasing attention has been directed toward the management of completely asymptomatic patients with vascular rings, whose number is increasing as a result of more frequent prenatal diagnosis. Currently, the prevailing view is that these patients do not require surgical intervention. However, arguments exist that consider waiting for the development of symptoms to be potentially risky. One reason is the assumption that complete correction by surgical intervention may not be possible after prolonged compression. Other arguments support correction regardless of the patient’s clinical status. Alternatively, early intervention may be preferred in young patients, in whom the tissues are more compliant, while surgical procedures in adult patients are more complex and associated with a higher operative risk [2].

### Limitations

The main limitations of this study are its retrospective design and small sample size, which may have influenced the results compared with those of larger patient cohorts.

## 5. Conclusions

In our study, we focused on vascular rings as a rare cause of respiratory symptoms, particularly in young children. It is important for healthcare professionals to recognize and identify their clinical manifestations, especially in patients who do not respond adequately to conventional treatment for recurrent respiratory infections, particularly when accompanied by gastrointestinal symptoms. We presented a small cohort of patients managed at our institution and compared our diagnostic process with other proposed diagnostic approaches. In this series, echocardiography was a useful non-invasive modality for raising suspicion and assessing associated cardiac abnormalities, whereas cross-sectional angiographic imaging was required to define vascular anatomy. In recent years, prenatal diagnosis has gained increasing importance and is substantially changing the approach to the diagnosis and management of vascular rings. Finally, our cohort included a rare combination of a vascular ring and a pulmonary artery sling in a single patient, a finding that remains highly uncommon to date.

## Figures and Tables

**Table 1:** Demographic and clinical characteristics of the cohort (*n* = 7).

Parameter	*n*	Median (IQR)	Range	Mean
Age at symptom onset (days)	7	30 (1–120)	1–5170	777.6
Age at definitive diagnosis (months)	7	14.5 (4.3–94.0)	1.25–185	56.8
Gestational age at birth (weeks)	7	38 (36.5–39)	32–40	37.3
Birth weight (g)	7	2600 (2048–3205)	1700–4030	2690.7
Age at surgery (months)	6	10.5 (4.1–20.1)	1.5–202	41.6
Diagnosis-to-surgery interval (days)	6	10 (7–160.8)	7–540	130.7

*n*, number of patients with available data (one patient did not undergo surgery); IQR, interquartile range (calculated using the inclusive method).

**Table 2:** Clinical symptoms in the cohort (n = 7).

Respiratory Symptoms	*n* (%)	Gastrointestinal Symptoms	*n* (%)
Cough	6 (85.7)	Dysphagia	5 (71.4)
Dyspnea	6 (85.7)	Recurrent vomiting	4 (57.1)
Stridor	5 (71.4)	Failure to thrive	2 (28.6)
Recurrent respiratory infections	5 (71.4)	Prolonged feeding	1 (14.3)
Cyanosis	4 (57.1)		
Apnea	2 (28.6)		
Wheezing	0 (0.0)		

Data are presented as number of patients (percentage). No patient was asymptomatic at the time of definitive diagnosis.

## Data Availability

The data presented in this study are available upon request from the corresponding author. The data are not publicly available due to ethical and privacy restrictions.

## Supplementary Materials

The following supporting information can be downloaded at https://www.mdpi.com/article/10.3390/life16101736/s1, Supplementary Table S1: Clinical characteristics, diagnostics, and treatment of patients with vascular rings.

## Abbreviations

The following abbreviations are used in this manuscript:

ARSA	Aberrant right subclavian artery
BAV	Bicuspid aortic valve
CHD	Congenital heart disease
CT	Computed tomography
CTAG	CT angiography
DAA	Double aortic arch
ICD-10	International Classification of Diseases, 10th Revision
IQR	Interquartile range
LPA	Left pulmonary artery
MRI	Magnetic resonance imaging
PAS	Pulmonary artery sling
PDA	Patent ductus arteriosus
RAA	Right aortic arch
RAA + ALSA	Right aortic arch with aberrant left subclavian artery
RPA	Right pulmonary artery
